# The long-term impacts of hearing loss, tinnitus and poor balance on the quality of life of people living with and beyond cancer after platinum-based chemotherapy: a literature review

**DOI:** 10.1007/s11764-022-01314-9

**Published:** 2023-01-13

**Authors:** Olivia R. Phillips, David M. Baguley, Stephanie E. Pearson, Michael A. Akeroyd

**Affiliations:** 1 Hearing Sciences, Mental Health and Clinical Neurosciences, School of Medicine, University of Nottingham, Nottingham, NG7 2RD UK; 2 NIHR Nottingham Biomedical Research Centre, Ropewalk House, 113 The Ropewalk, Nottingham, NG1 5DU UK; 3 The University of Nottingham Health Service, Cripps Health Centre, University Park, Nottingham, NG7 2QW UK

**Keywords:** Ototoxicity, Cancer, Quality of life, Survivorship, Long-term effects, Hearing loss, Tinnitus

## Abstract

**Purpose:**

To elucidate the long-term impacts of hearing loss, tinnitus and balance in people living with and beyond cancer (LWBC) treated with platinum-based chemotherapy (PBCT).

**Methods:**

A literature search was conducted between March and June 2022 using PubMed, Web of Science and Google Scholar. Full-text papers in English were included. Articles explored the impacts of hearing loss, tinnitus and balance and discussed them in the context of treatment. If PBCT was used in conjunction with other treatments, the article was included. There were no constraints on age, cancer type, publication date, location, study design or data type. Sixteen studies and two reviews were included.

**Results:**

Hearing loss and tinnitus can cause communication difficulties and subsequent social withdrawal. There were deficits in cognition, child development and educational performance. Employment and the ease of everyday life were disrupted by hearing loss and tinnitus, whereas poor balance interfered with walking and increased the risk of falls. Depression and anxiety were related to ototoxicity. Most notable were the differing mindsets experienced by adults LWBC with ototoxicity. There was evidence of inadequate monitoring of ototoxicity by clinicians and a lack of communication between clinicians and patients about ototoxicity as a side effect.

**Conclusions:**

Ototoxicity has a negative long-term impact on multiple areas of life for adults and children LWBC. This can compromise their quality of life.

**Implications for cancer survivors:**

Increased awareness, monitoring and education surrounding these issues may lead to earlier intervention and better management of ototoxicity, enhancing the quality of life of people LWBC.

**Supplementary Information:**

The online version contains supplementary material available at 10.1007/s11764-022-01314-9.

## Introduction

To be diagnosed with cancer is a life-changing and life-threatening event, which can naturally evoke feelings of fear and uncertainty in patients. In 2020, there were approximately 20 million new cases of cancer and almost 10 million deaths worldwide [1]. However, due to medical advances, an increasing number of people are now surviving. For example, in the USA, the 5-year survival rate for all cancers combined has improved from 49 to 68% between 1975 and 2017 [2]. Although survival is the main aim of cancer treatment, these increasing rates indicate that more people are living with the long-term effects of treatment, which can negatively impact the quality of life of patients in remission [3]. Although ‘quality of life’ is a broad and multi-faceted concept with various definitions, within this review it is interpreted as the perception of an individual’s well-being, or lack of, on a daily basis [4, 5]. It is imperative to understand the long-term effects experienced by those living with and beyond cancer (LWBC) so that measures can be implemented to ameliorate and manage them. In this article, we are concerned about the lasting impacts of hearing loss, tinnitus and poor balance after cancer treatment.

Solid tumours, such as ovarian, breast and testicular cancers, as well as head, neck and non-small cell lung cancers, are often treated with platinum-based chemotherapies (PBCT), namely cisplatin, carboplatin and oxaliplatin [6]. Although these chemotherapeutic agents are highly effective, they are not without their disadvantages. It is well established that PBCT, especially cisplatin, are ototoxic, meaning they cause damage to the inner ear structures like the cochlea [7]. This can result in conditions such as tinnitus and hearing loss [8]. The outer hair cells in the basal turn of the cochlea are most susceptible to damage, causing high frequencies to deteriorate first (typically above 8 kHz) [9] followed by progressively lower frequencies as treatment continues [10]. The severity of this hearing loss has been shown to be cumulative and dose-dependent, with the effects being bilateral and permanent [11]. Similarly, radiotherapy can cause hearing loss through damage to auditory structures. For example, lesions of the Eustachian tube or osseous chain in the middle ear can cause conductive hearing loss and lesions of the cochlea/retro-cochlea regions can cause sensorineural hearing loss [12]. ‘Ototoxicity’, however, refers not only to damage of the hearing apparatus but also to damage of the vestibular labyrinth, important for maintaining balance [13]. Damage to this inner ear structure can therefore result in general postural instability, a greater risk of falls and consequent injury [14]. Together, this research presents a difficult scenario faced by people LWBC. That being, whilst the chances of survival may increase with continued treatment, this also increases the risks of impaired audio-vestibular functioning.

Within the general population, hearing loss has been related to social isolation, loneliness, mental health problems i.e., depression, as well as cognitive dysfunction such as dementia [15]–[17]. Similarly, tinnitus is associated with heightened anxiety, depression, insomnia and reduced concentration [18]–[20]. There is relatively less research related to hearing loss and tinnitus in those LWBC, however, despite the estimated prevalence of ototoxicity in both adults and children being greater than 50% [21]. There is even less research related to vestibulotoxicty in this group, such as poor balance [22]. Furthermore, unlike the general population, those LWBC have undergone the traumatic experience of being diagnosed with cancer and receiving treatment. Individuals may live most of their life with normal hearing, which then becomes compromised. There is evidence that this can happen after just one cycle of cisplatin [23], which can have a negative effect on quality of life. For example, a qualitative research study and a narrative review have demonstrated that adults LWBC who experience hearing loss, tinnitus and poor balance have a poorer quality of life compared to comparison populations [3, 24]. This highlights the necessity to direct more of our attention towards this population to improve care provision and help individuals adapt to life after cancer.

This literature review aims to serve as an update on the current literature and research on this topic. We aim to collect and summarise what is known on how hearing loss, tinnitus and poor balance affect the quality of life of people LWBC who have undergone PBCT. Although increasing, this is currently an understudied research area. For the purposes of conciseness, the focus will remain on PBCT only or PBCT in combination with other treatments i.e., radiotherapy. It will not discuss the extent of hearing loss, tinnitus etc. in those LWBC but rather the wider impacts of these on quality of life. Issues regarding the monitoring of ototoxicity and the lack of patient awareness of this as a treatment side effect will also be discussed.

## Methods

Literature searches were conducted between March and June 2022 using PubMed, Web of Science and Google Scholar. The keywords, ‘cancer’, ‘survivor’, ‘chemotherapy’, ‘platinum’, ‘cisplatin’, ‘carboplatin’, ‘oxaliplatin’, ‘ototoxicity’, ‘vestibulotoxicity’, ‘hearing loss’, ‘tinnitus’, ‘balance’, ‘long term’ and ‘quality of life’ were inputted in various combinations using Boolean operators (see Online Resource 1 for details on the search strategy).

**Fig. 1:**
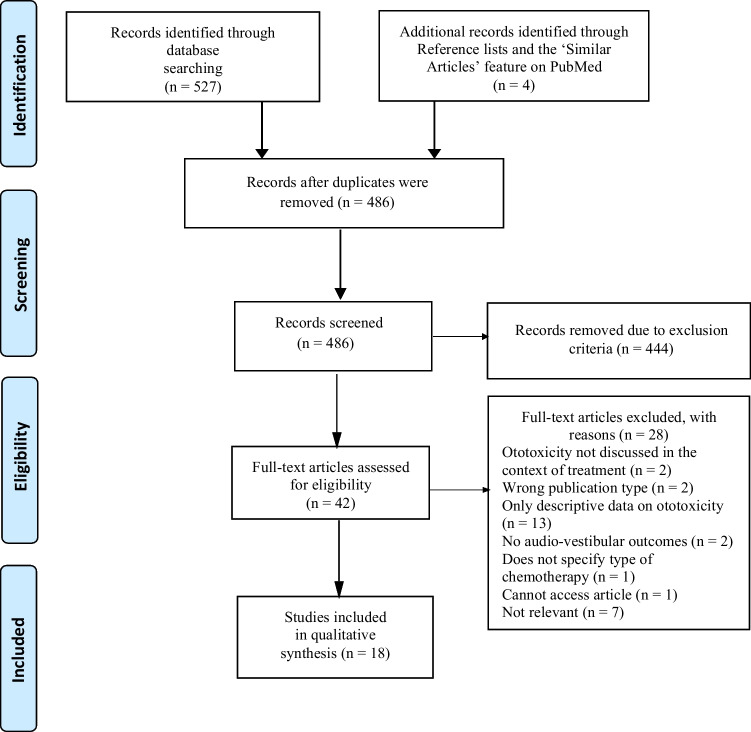
Process of selection of studies for this literature review based on the PRISMA flow diagram

To facilitate a complete and holistic understanding of the topic, few constraints were imposed and the search remained broad. There were no limitations on publication date, location, study design or data type (i.e., quantitative, qualitative or mixed). Reviews were included if they referred to the impacts of audio-vestibular dysfunction in relation to cancer treatment. We focused on published literature only; therefore, grey literature was excluded. No age constraints were applied, meaning articles concerned both adults and children treated for any type of cancer. Pre-clinical and in vitro studies were excluded. Only clinical data published as full-text papers in English were included. The articles must have explored the impacts of hearing loss, tinnitus and poor balance as a primary or secondary outcome and discussed them in the context of treatment. Articles that simply described the prevalence of ototoxicity after treatment were excluded. Given that our focus is on PBCT, articles concerning the use of other treatments alone (i.e., radiotherapy) were also excluded. However, if chemotherapy was used in conjunction with other treatments, the article was included. The initial searches found 527 titles. After removing duplicates (*n* = 41), 486 titles remained. This number was then reduced through title and abstract screening by the primary author, OP (*n* = 42). Full-text reading by the primary author reduced this further, according to the inclusion/exclusion criteria (*n* = 14). However, four additional articles found by manually searching reference lists (*n* = 3) and through the ‘similar articles’ feature on PubMed (*n* = 1) were also included. The total number of included titles was, therefore, 18. This process is summarised in Fig. 1.

## Results

Of the 18 titles accepted, 16 of them were individual studies and two of them reviews. In total, the number of participants included in the studies of this literature review sum to 12,210 (quantitative studies: sum = 4531, range = 12–1520, median = 165; qualitative studies: sum = 421, range = 20–377, median = 24; reviews: sum = 7258, range; 1–623, median, 3629). The included studies have been summarised in Tables 1, 2 and 3. These report the overall characteristics of the datasets; however, the results are described and contextualised in the ‘Discussion’ section.

**Table 1:** A summary of the included quantitative studies

Author (year)	Study type	Cancer type	Treatments	Number of participants	Number of patients receiving PBCT	Follow-up	Patient characteristics	Outcome measures			Control
								**Hearing loss**	**Tinnitus**	**Balance**	
Aggarwal et al. (2022)	Cross-sectional, self-report	OPC	Surgery, neck dissection, RT, chemotherapy, concurrent cisplatin therapy	880	95 concurrent high-dose cisplatin, 122 concurrent low-dose cisplatin, 19 other concurrent cisplatin	Completed ≥ 1 year of post-treatment follow-up	Adults (age at diagnosis ≥ 18 years); received curative OPC treatment during January 2000–December 2013. Median survival time 7 years (range 1–16 years). Median age at diagnosis 56 years (range 32–84)	MDASI-HN survey: respondents rate their ‘difficulty with hearing loss and/or ringing in the ears’ from 0 (not present) to 10 (as bad as you can imagine)	MDASI-HN survey: respondents rate their ‘difficulty with hearing loss and/or ringing in the ears’ from 0 (not present) to 10 (as bad as you can imagine)	Not measured	Comparison with patients reporting no hearing loss or tinnitus (*n* = 313)
Al-Khatib et al. (2010)	Prospective cohort study, self-report	HL, NHL, OS, RB, OG, NPC, abdominal, genital, brain	PBCT and RT	31 (only 21 included in the long term follow up)	18 cisplatin, 10 carboplatin, 3 both	Median time since diagnosis 3.4 years	Patients diagnosed between 2000 and 2005. Average age at diagnosis, 8 years (range; 5 months – 17 years). The minimum ototoxic dose was 302.06 mg/m^2^	Audiometer, bone conduction thresholds, otoacoustic emission testing	Not measured	Not measured	Not measured
Bass et al. (2020)	Cross-sectional	ALL, AML, HL, NHL, WT, NB, RB, GCT, HB, OS, RMS, EW, NRSTS, CNS tumour, other	PBCT, cochlear RT, cranial RT, other	1520	307 cisplatin and/or carboplatin only, 473 cisplatin/carboplatin with RT	Median time since diagnosis 20.35 years	Patients who had participated in the St Jude Lifetime Cohort Study. Mean age at diagnosis was 6.57 years. Median age at study was 29.40 years. Survived ≥ 5 years after original diagnosis. Cumulative median dose carboplatin 2821.4 mg/m^2^. Cumulative median cisplatin dose 400 mg/m^2^	Otoscopy, tympanometry, pure tone audiometry, speech audiometry, air conduction and bone conduction	Not measured	Not measured	Cochlear RT exposure group (*n* = 473) and no exposure group (*n* = 740)
Brinkman et al. (2015)	Cross-sectional, self-report	MB, OS, GCT, NB, RMS, NPC, ependymoma, astrocytic tumours, other brain tumours	Cranial RT, PBCT	406	211 cisplatin, 39 carboplatin	Mean years since diagnosis: 10 ≤ years > 40	Patients who had participated in the St Jude Lifetime Cohort Study. Survived ≥ 10 years since original diagnosis of a CNS or non-CNS tumour. Mean age at diagnosis 8.40 years (CNS), 8.7 years (non-CNS). Mean age at follow-up 26.6 years (CNS), 31 years (non-CNS)	Pure-tone air conduction, bone conduction, tympanometry	Not measured	Not measured	Patients without severe hearing loss (CNS, *n* = 115; non-CNS, *n* = 137)
Einarsson et al. (2010)	Longitudinal observational	OS, ST, ET, CO, OT, MMT, NB, EW	PBCT	15	All received cisplatin	Median time since last cisplatin dose 16 years (hearing impaired), 10.40 years (normal hearing)	Median age at study, 27.50 years (hearing impaired), 23.70 years (normal hearing). Median age at diagnosis, 10.30 years (hearing impaired), 15.70 (normal hearing), (range; 0.1–17 years). All subjects had normal hearing between 0.125 and 8 kHz pre-chemotherapy. Median cumulative cisplatin dose 480 mg/m^2^ (hearing impaired), 360 mg/m^2^ (normal hearing)	Pure-tone audiometry, tympanometry, speech audiometry, HMS questionnaire	HMS questionnaire	Not measured	Patients without hearing loss (*n* = 9)
Gurney et al. (2007)	Cross-sectional, self-report	NB	PBCT, other non-platinum agents, surgery, bone marrow transplantation	137	54 with cisplatin and other agents, 25 with cisplatin, carboplatin and other agents	Mean years since diagnosis, 11.10	Mean age at diagnosis 1.4 years, mean age at interview, 12.10 years (range: 8–17 years). Previously enrolled in one of two NB clinical studies. Patients classified as low, intermediate or high-risk	Parents completed instrument designed by clinical study team, ‘Have you been told by a doctor or other health care professional that your child, who had neuroblastoma, currently has (or had in the past) any of the following’	Not measured	Not measured	NB patients without hearing loss (*n* = 94)
Miaskowski et al. (2018a)	Cross-sectional, self-report	Breast, colon, lung, ovarian, other	PBCT, taxane compound, combination of PBCT and taxane compound	371	53 cisplatin, 111 carboplatin, 52 oxaliplatin	Years since diagnosis: CIN, 4.09; CIN and hearing loss, 5.98; CIN, hearing loss and TIN, 5.60	Mean age at study CIN 59.68 years; CIN, hearing loss 64.13 years; CIN, hearing loss, TIN; 62.50 years. Mean dose of PBCT for patients who received only PBCT 678 mg/m^2^	FACT/GOG-Ntx	FACT/GOG-Ntx	CIPNAT, TUG and FAB	Comparison with patients with CIN only (*n* = 217), CIN and hearing loss (*n* = 69) and CIN, hearing loss and TIN (*n* = 85)
Miaskowski et al. (2018b)	Cross-sectional, self-report	Breast, colon, lung, ovarian, other	PBCT, taxane compound, combination of PBCT and taxane compound	195	59 PBCT, 41 PBCT and taxane compound	Years since diagnosis: no neurotoxicity group, 4.60; neurotoxicity group, 5.60	Mean age at study: no neurotoxicity groups, 57.30 years; neurotoxicity group 62.50 years. Mean dose of PBCT for patients who received only PBCT 554.62 mg/m^2^	FACT/GOG-Ntx	FACT/GOG-Ntx	CIPNAT, TUG and FAB	Comparison with patients without neurotoxicity (*n* = 110)
Niederer et al. (2014)	Cross-sectional, self-report	Mainly breast cancer (81%)	PBCT, taxane-based chemotherapy, combination of PBCT and taxane-based	21	7 PBCT (type not specified), 8 taxane-platinum combination	Median time since diagnosis, 9 months (range 2–24)	Cancer patients who had recently completed treatment < 12 months ago. Twenty females and one male. Age 50 ± 7	Not measured	Not measured	Capacitive force-measuring platform (30 Hz) WinFDM v.0.0.41	Age-matched healthy controls (*n* = 24) and senior controls (*n* = 24)
Rajput et al. (2020)	Retrospective cross-sectional, self-report	Brain tumours, eye tumours, non-brain solid tumours, lymphoma, leukaemia	PBCT, a combination of PBCT and RT, none	78	29 carboplatin, 12 cisplatin, 6 carboplatin and cisplatin, 16 PBCT and RT	Mean years since diagnosis; hearing loss group 3.56, normal hearing group, 4.27	Children aged 5–18 at data collection, treated with chemotherapy for any cancer type. Age at diagnosis: hearing loss group 5.90 years; normal hearing group 5.81 years. Age at study: hearing loss group 9.46 years, normal hearing group 10.08 years	Pure-tone audiometry	Not measured	Not measured	Patients with normal hearing (*n* = 36)
Schreiber et al. (2014)	Longitudinal	MB	Craniospinal irradiation, surgery chemotherapy with stem cell support	165	All received cisplatin	Baseline, 1, 3 and 5 years post diagnosis	Patients aged 3–21 at diagnosis, mean age at diagnosis 9.88 years, classed as average or high risk. Cumulative cisplatin dose 300 mg/m^2^	Tympanometry, pure-tone air and bone conduction thresholds, distortion product otoacoustic emissions, auditory brainstem response, auditory steady-state response	Not measured	Not measured	No control
Waissbluth et al. (2018)	Prospective cohort study, self-report	MB, OP/HG, HB, NB, germinoma (hypothalamic), astrocytoma, frontal OT, ovarian OT	PBCT and cranial irradiation	12	7 cisplatin, 2 carboplatin, 3 both agents	Median follow up of 11.9 years post chemotherapy	Median age at beginning of chemotherapy 4.30 years (range; 10 months–14.20 years). Received PBCT between 2001– and 2006. Cisplatin cumulative dose; 506.40 ± 232 mg/m^2^2. Carboplatin cumulative dose, 2093 ± 691 mg/m^2^	Tympanometry, pure-tone air and bone conduction thresholds, SSQ12	Not measured	Not measured	Comparison with patients without hearing loss (*n* = 6)
Weiss et al. (2018)	Cross-sectional, self-report	NB, RB, GCTs, CNS tumours leukaemia, lymphoma, renal tumours, hepatic tumours, bone tumours, soft tissue sarcomas, other	Surgery, cranial RT, chemotherapy, bone marrow transplantation	700	150 PBCT, type not specified	Survived ≥ 5 years after initial diagnosis	Mean age at diagnosis; CNS tumours, 5 years; non-CNS tumours, 3 years. CNS group 50% female, non-CNS group 42% female	Non-validated questionnaire asking parents if a doctor has told them their child has hearing loss	Not measured	Not measured	Patients with CNS and non-CNS tumours without hearing loss (*n* = 631)

Abbreviations: *ALL*, acute lymphoblastic leukaemia; *AML*, acute myeloid leukaemia; *CIN*, chemotherapy-induced neuropathy; *CIPNAT*, chemotherapy-induced peripheral neuropathy assessment tool; *CNS*, central nervous system; *CO*, chondroblastic osteosarcoma; *ET*, embryonal teratoma; *EW*, Ewing sarcoma; *FAB*, Fullerton Advanced Balance Test; *FACT/GOG-Ntx*, Functional Assessment of Therapy/Gynaecologic Oncology Group Neurotoxicity; *GCT*, germ cell tumour; *HB*, hepatoblastoma; *HL*, Hodgkin’s lymphoma; *HMS*, hearing measurement scale; *MMT*, malignant mixed tumour; *MB*, medulloblastoma; *MDASI-HN*, MD Anderson Symptom Inventory Head and Neck Cancer Module; *mg/m*^*2*^, milligrams per m^2^; *NB*, neuroblastoma; *NHL*, non-Hodgkin’s lymphoma; *NPC*, nasopharyngeal carcinoma; *NRSTS*, non-rhabdomyosarcoma soft tissue sarcoma; *OG*, optic glioma; *OP/HG*, optic pathway/hypothalamic glioma; *OPC*, oropharyngeal cancer; *OS*; osteosarcoma; *OT*, immature teratoma; *PBCT*, platinum-based chemotherapy; *RB*, retinoblastoma; *RMS*, rhabdomyosarcoma; *RT*, radiotherapy; *SSQ12*, Speech, Spatial and Qualities of Hearing scale (abbreviated version); *ST*, sacrococcygeal teratoma; *TIN*, tinnitus; *TUG*, Timed Get Up and Go test; *WT*, Wilms tumour

**Table 2:** A summary of the included qualitative studies

Author (year)	Methodology	Participants	Cancer type	Treatments	Data analysis
Kahn et al. (2020)	Semi-structured telephone interviews. Participants were asked about life with hearing loss, hearing aids and their interactions with audiologists/clinicians	24 adult cancer patients with hearing loss, evidenced by clinical notes, audiograms or the use of hearing aids. Treated < 40 years of age. Recruited from the Adult Long-Term Follow-Up Program at Memorial Sloan Kettering Cancer centre established in 2005	Most common cancer types include leukaemia, lymphoma, brain tumours, sarcoma, hematopoietic stem cell transplantation	Exposure to cranial radiotherapy, platinum chemotherapy or both	Interviews were transcribed verbatim, then read and coded by 2 independent investigators for inductive thematic analysis. Three investigators then organised the data into themes and identified suitable quotations
Pearson et al. (2019a)	Inductive thematic analysis of 9 publicly available, English, online health care forums. A total of 86 threads were included. The forums were from Google, Bing Yahoo! and AOL. Posts discussed hearing loss or tinnitus and cancer or chemotherapy	377 cancer patients who contributed to the health care forum threads	Data not collected	Cisplatin, carboplatin, oxaliplatin and non-platinum drugs	Whole threads were screened, and only those deemed relevant were used. Open coding of first 100 messages to create a pilot coding manual. Two independent researchers coded the remaining messages against the manual and changed it accordingly, until a final coding manual was agreed on and thematic analysis could occur
Pearson et al. (2021)	In person semi-structured interviews (in conjunction with taking field notes) investigating the impacts of ototoxicity on quality of life, awareness of ototoxicity as a side effect and what support has been offered	20 cancer patients who had self-reported or diagnosed hearing loss/tinnitus ≥ 6 months post-chemotherapy. Mean age 53.10. Median years since chemotherapy 4.50	Stomach, breast, testicular, multiple myeloma, bowel, leukaemia, cervical	Chemotherapy (7 cisplatin, 3 carboplatin, 1 oxaliplatin, 5 unknown, 4 other)	Interviews transcribed verbatim before inductive thematic analysis was conducted. Thirty-four codes developed from the transcripts and field notes. Through discussion with a second researcher, these codes were grouped into subthemes and themes

**Table 3:** A summary of the included reviews

Author (year)	Publication dates of included studies	Total number of studies	Total number of participants	Type of review
Wechsler and Wood (2021)	2007–2020	30	*n* = 4778. Median sample size for 29 studies, 59 (range; 1–623). One study was excluded from the aggregation of clinical characteristics and participants as it was an outlier in almost all categories (*n* = 65,311)	Scoping review
Wang et al. (2021)	2001–2020	32	From the data available from 24 studies, *n* = 2480, range (8–512)	Quantitative narrative review

Table 1 summarises the 13 quantitative studies, which often included participants with a variety of cancer types diagnosed when they were children (< 18 years). Mean age at cancer diagnosis across all patients in these studies was 22.11 years. The range of ages at diagnosis could not be calculated as only 5/13 studies provided this data. Where they did, this is included in the relevant ‘Patient characteristics’ section in Table 1. However, using the data available, the range of age at diagnosis is 1.2 months–84 years. The total number of participants is *n* = 4531 (range; 12–1520). All studies included patients who had received PBCT, with cisplatin being the most common, however some patients also underwent alternative treatments either in conjunction with PBCT or instead of PBCT. These included surgery (*n* = 4/13), neck dissection (*n* = 1/13), radiotherapy (*n* = 9/13), taxane-based chemotherapy (*n* = 3/13), bone marrow transplantation (*n* = 2/13), and chemotherapy with stem cell support (*n* = 1/13). Twelve out of 13 studies measured hearing loss, however the measurement of tinnitus and balance was much less common (*n* = 5/13, *n* = 3/13, respectively). The type of follow-up varied, with ten studies reporting the time since diagnosis and three reporting the time since the last dose of treatment. Follow-up ranged from just 2 months to over 40 years. Measurements included a variety of subjective and objective methods. For example, hearing loss was measured using objective methods such as otoacoustic emissions (*n* = 2/13), bone conduction thresholds (*n* = 5/13), air conduction thresholds (*n* = 6/13), tympanometry (*n* = 5/13), otoscopy (*n* = 1/13), speech audiometry (*n* = 2/13) and auditory steady-state responses (*n* = 1/13). Hearing loss was also measured using subjective methods, such as SSQ12 (*n* = 1/13), MDASI-HN (*n* = 1/13) and questionnaires created by the research team (*n* = 2/13). Tinnitus was measured using the HMS questionnaire (*n* = 1/13), MDASI-HN survey (*n* = 1/13) and FACT/GOG-Ntx (*n* = 2/13), whereas measurements of balance included CIPNAT, TUG, FAB (all *n* = 2/13) and the WinFDM platform (*n* = 1/13). In six studies, participants were children. Three of these included parent-reported outcomes and two included child-self-reported outcomes. Nine studies had a control group which compared those LWBC with hearing loss to those without.

Table 2 summarises the three qualitative studies in this review. The total number of participants is *n* = 421 (range; 20–377). Two studies used semi-structured interviews to collect primary data, whereas one analysed secondary data in the form of online healthcare forums. Within these forums, those LWBC suffering from the effects of treatment discuss how ototoxicity impacts their quality of life. All three studies used thematic analysis. Only one study explicitly stated the age of participants, another described them as ‘adults’ and it is assumed through their use of online healthcare forums that participants in the remaining study were also adults. Two studies provided data on cancer type. PBCT was used in all three studies, however treatments also included non-platinum agents and radiotherapy.

Table 3 summarises the two included reviews. Together, they spanned literature from 2007 to 2020. Both focused on balance after chemotherapy, rather than hearing loss or tinnitus. The total number of participants included in the papers of these reviews summed to *n* = 7258 (range; 1–623). This excludes one outlier from Weschler and Wood (2021) [25], with 65,311 participants. This figure also accounts for the papers included by both reviews (*n* = 13) and for missing data. The review by Wang et al. (2021) [24] only included the number of participants for 24/32 of the included studies. The author was contacted about this; however, we received no response. The total number of participants included in these reviews excludes this missing data, therefore it is not completely accurate.

## Discussion

This literature review aimed to highlight the challenges faced by those LWBC after treatment. During the literature search, it became apparent that whilst hearing loss is relatively well researched in cancer populations, there are fewer publications on the effects of tinnitus and poor balance. Despite this, there were several clear themes concerning the long-term impacts of treatment-induced ototoxicity, supported by evidence in this review. These long-term impacts are of a social and cognitive nature. It can also have severe impacts on the development of children and their later academic performance. Employability prospects are affected as well as the ease of completing simple everyday tasks. Finally, ototoxicity can also result in mental health problems. These themes will be discussed in turn below.

## Social impacts

A commonly reported impact of hearing loss after PBCT is the inability to perceive and hear speech. This renders communication with others, therefore participation in social events or group conversations, extremely challenging. For example, a qualitative study of 24 adults of childhood/young adult cancer found that hearing loss discourages those LWBC from participating in group conversations, with some reporting that they nod along or pretend to know what has been said when actually, they have not heard [26]. Other barriers to group conversation arise out of fear of misunderstanding what has been said, having to explain their cancer diagnosis, or having to ask strangers, who are unaware of their diagnosis, to repeat themselves. These findings are concordant with both a prospective cohort study and a longitudinal observational study showing that those LWBC, treated with PBCT, believe their largest handicap lies within speech hearing [27, 28]. For these patients, it had been an average of 12 and 16 years since their chemotherapy, demonstrating that the effects of hearing loss remain well into adulthood. This inability to perceive speech can cause feelings of embarrassment and a dislike of speaking in front of others [26, 28]. Furthermore, speech localisation after receiving PBCT is also impaired, which again makes participation in group conversation extremely difficult [27]. An issue that must be considered whilst interpreting this research, however, are the small sample sizes, Waissbluth et al. (2018) [27] had 12 participants, and Einarsson et al. (2010) [28] had 15. In both studies, there were only 6 hearing impaired participants. Although small sample sizes do not always correlate to the quality of research, a rationale as to why a small sample size was used must be reported. For example, a pilot or feasibility study does not need a power analysis. These studies, however, did not report a rationale as to why a small sample size was used. Similarly, Kahn et al. (2020) [26] had only 24 participants. It is typical in qualitative research to have sufficient participants to ensure data saturation; however, there was no mention of saturation; therefore, it remains uncertain whether it was achieved.

It is not only hearing within a group that is problematic. Hearing outdoors, whilst travelling, or even watching television can also be difficult [27, 28]. Generally, hearing in a noisy environment, hearing quiet sounds or hearing sounds amongst background noise is challenging for those with treatment-induced hearing loss [26]–[29]. Speech in the high pitch range, for example that of women and children, can be particularly unintelligible. In a qualitative study by Pearson et al. (2021) [29] where approximately half of interviewees received PBCT, one reported, ‘I just couldn’t hear, especially if people spoke softly, or women and children’s voices. I just couldn’t hear them’ [29]. This lack of intelligibility may be associated with the frequencies of specific phonemes in the English language. For example, fricative phonemes (e.g., /*f*/, /*s*/, /*v*/), which make up 50% of English consonants, rely on the perception of high frequencies. However, these are the frequencies initially lost after ototoxic cancer treatments, which makes distinguishing between phonemes challenging, even with mild hearing loss [30].

As with hearing loss, tinnitus also impedes communication and causes frustration in those LWBC. For example, someone reported, ‘I’m knackered and it’s just hiss. People can stand in front of me and speak and I’m stressing because I just hear hiss’ [29]. Another reported being unable to engage in anything due to tinnitus. These barriers to communication have been shown to effect relationships with partners and family members. Compared to those without tinnitus and hearing loss, those with mild or moderate-severe hearing loss and tinnitus had greater odds of self-reported interference in relations with others [31]. This demonstrates that the degree of ototoxicity does not even have to be severe to cause a negative impact. However, it must be noted that in this cross-sectional study, participants were asked to self-report their ‘difficulty with hearing loss and/or ringing of the ears at its worst’. Here, hearing loss and tinnitus, two separate long-term effects of cancer treatment, are measured together. This renders it impossible to tease out the impacts of these individually. This is important to do since overcoming the barriers of hearing loss may not be the same as overcoming the barriers of tinnitus. Each may require tailored and different interventions. There was also no information on pre-treatment hearing assessments, which makes it difficult to distinguish the effects of the tumour versus the effects of the treatment on hearing function. Despite these issues, it appears that ototoxicity is linked to negative relations with others. This is supported by an analysis of online healthcare forums, where one user describes that their tinnitus drives them mad and is now affecting their relationship [32]. This has wide-reaching consequences. Ototoxicity can impact people’s relationships often in times of increased social need.

The above barriers to communication can cause social anxiety and withdrawal from situations associated with this anxiety [26, 28, 33]. Cross-sectional research has demonstrated that adults treated for childhood cancer, who went on to develop severe hearing loss, are more likely to spend their leisure time on computers or watching television—relatively isolating activities [33]. Others reported that their hearing loss makes them feel reluctant to join social events [28], as well as avoid crowded rooms out of fear they would have to be at an uncomfortably close range to someone to hear them speak [26]. Since hearing loss is often described as an invisible condition [34], these social challenges are not necessarily recognised by others.

Communicating with others is not the only measure of impaired social attainment after PBCT. This can extend to other facets of life too. For example, in a sample of 226 adults of non-CNS (central nervous system) cancers where it had been at least 10 years since diagnosis, Brinkman et al. (2015) [33] found that participants with severe hearing loss had an increased risk of not living independently compared to those without severe hearing loss. This particular life event promotes self-reliance and socialisation and represents a sense of adulthood, achievement and individuality. Although only one measure of social functioning, this may highlight the lack of autonomy possessed by some adults LWBC with hearing loss. It also demonstrates the decreased opportunity to integrate into the community. However, the same pattern was not seen for patients of CNS cancers with hearing loss, who showed an increased, although not statistically significant, risk of not living independently.

Together, the above research suggests that ototoxicity after cancer treatment permeates many facets of social functioning in later life. It increases barriers to social support, decreases the size of social networks and restricts community integration. This is especially problematic for those LWBC, who may require greater support and reliance on others. In addition to other long-term and late effects and how these comorbidities impact an individual, the addition of ototoxicity can impact a person’s independence. Given that humans are a naturally social species, perceived social isolation has negative repercussions on several health outcomes, such as mental health, cognition, physical health and all-cause mortality [35, 36]. This highlights the importance of increasing awareness of post-chemotherapeutic social isolation. To further combat social difficulties, hearing interventions, such as hearing aids, should be used. Hearing aids are the most commonly implemented intervention for presbycusis in adults [37] and although they do not restore hearing to normal, they have been shown to improve quality of life [38] and decrease feelings of loneliness [39]. Most manufacturers now design them to be compatible with a range of devices, such as televisions, radios and phones [40]. However, there is evidence that hearing aids are underused in cancer populations [8, 33], with one study showing that only one-third of adults LWBC with hearing loss used a hearing intervention [33], although, due to the cross-sectional nature of this study, which provided only a snapshot of time, we cannot be sure the uptake of hearing interventions did not improve, highlighting the need for more longitudinal research. Nevertheless, this is a potential research question which should be investigated further. What are the barriers to the uptake of hearing interventions? Is the poor uptake due to patients or clinicians? These questions are becoming increasingly important given the growing number of people LWBC with hearing loss—an invisible yet potentially restrictive and isolating disability.

## Cognitive impacts

Ototoxicity has also been demonstrated to negatively affect the cognitive abilities of those LWBC. Cognition is a broad term encapsulating processes such as attention, memory, executive function, processing speed and language [41]. Cross-sectional research studies have demonstrated that adults LWBC with hearing loss are more susceptible to cognitive decline than those without. For example, even after adjusting for relevant covariates and treatment dosage, there is evidence that compared to those with normal or mild hearing loss, adults LWBC with severe hearing loss are at a greater risk of impaired performance on language dependent tasks [42]. This includes verbal reasoning, verbal fluency and word reading. Mathematical computation was also worse. These patients had a median age of 5 years at diagnosis and were followed-up at age 27. That this data were gathered more than 20 years since patients were diagnosed highlights the permanence of hearing loss after treatment as well as its long-term effect on cognitive performance. This study combats the earlier issue of smaller sample sizes, with this sample being composed of 1520 adults treated for childhood cancer. However, given its cross-sectional nature, we cannot know the onset of these cognitive deficits, which is important for early detection and intervention. This again suggests that more longitudinal designs, and continuous monitoring, are needed. Despite these limitations, there is supporting evidence from a longitudinal study of 165 medulloblastoma patients who had seven domains of cognitive ability tested [43]: verbal comprehension, visual-auditory learning, concept formation, visual matching, sound blending, spatial relations and numbers reversed. Results demonstrated that those with severe hearing loss had general intellectual abilities that were below average four years after diagnosis. In comparison, those without severe hearing loss did not. This may have negative consequences for academic performance. For example, severe hearing loss has been associated with a twofold increased risk of failing to graduate high school or being unemployed [33]. It is possible that these outcomes are mediated by cognitive decline, however this must be interpreted with caution as this relationship was not directly tested.

Cognitive deficits have also been observed for tasks which are less dependent on language. Compared to patients with normal or mild hearing loss, those with severe hearing loss show deficits in attention, processing speed, executive function and cognitive flexibility. They also have slower visuomotor speed [42]. This is supported by Miaskowski et al. (2018b) [44], who found that adults LWBC with neurotoxicity (damage to the central or peripheral nervous system) self-reported worse attentional functional scores at least 3 months after completion of treatment. Important to note is that here, ‘neurotoxicity’ is comprised of hearing loss, tinnitus and chemotherapy-induced neurotoxicity. Analyses do not separate these conditions therefore it is not possible to know which one(s) have the strongest association with cognitive deficits. Despite this, there is evidence that mild hearing loss can also impact cognition. When comparing the cognition of individuals LWBC who had normal hearing with those with mild hearing impairment, Bass et al. (2020) [42] demonstrated that those with mild hearing loss exhibited up to a 2.5 times increased risk for neurocognitive dysfunction. This included the domains of intelligence, attention, executive function and processing speed. Again, this displays that hearing loss does not have to be severe to impact the quality of life of people treated for cancer—a pattern we have seen before when discussing social difficulties after treatment-induced ototoxicity.

This decline in cognitive abilities in these quantitative studies are supported by qualitative data too. Using semi-structured interviews, adults LWBC complained of ‘chemo-brain’. Interviewees referred to losing things and feeling disorganised [29]. Others have also said that they are slower at taking in, processing and synthesising information because of their hearing loss. In fact, this has become a burden. One individual discussed the increased energy they now require to pay attention [26]. Together, this qualitative and quantitative data suggest a cognitive deficit related to hearing loss after cancer treatment. However, there are other important factors to consider which may also be affecting cognition, such as neuropathy and fatigue. These can also have long lasting and negative impacts on quality of life, making day to day life more challenging. Level of independence may be impacted, as well as decision-making and functional abilities. To slow cognitive decline which may be related to ototoxicity, however, hearing and cognitive assessments should be administered during cycles of chemotherapy as well as after. This is so early intervention and can occur and the identification of those patients at risk of cognitive decline.

## Child development and educational impacts

To experience post-chemotherapeutic ototoxicity has deleterious consequences for those LWBC, however these are especially wide-reaching and long-lasting for pediatric patients who are in a critical stage of their speech and language development. Given that mechanisms of word learning include repeated verbal cues [45] as well as children having the opportunity to monitor their own speech, a hearing deficit can cause a delay in language acquisition. For example, using a prospective cohort study design, speech alterations have been noticed by family members of pediatric patients an average of 3 years post-diagnosis [45]. This could be attributed to their lessened ability to hear others and to monitor themselves. Moreover, aside from vocabulary, the general rules of language and syntax are also learned through verbal cues [45]. As mentioned previously, the perception of fricative sounds, which make up 50% of English consonants, rely on the perception of high frequencies [30] but these are often the first to deteriorate after PBCT. High frequency sounds are also essential for identifying the plural marker /*s*/, approximately the third most frequently occurring consonant, as well as for determining tense and sex [30]. The importance placed on these frequently used sounds can result in errors when perceiving and learning language, and can manifest as educational, social and emotional difficulties.

The impaired ability to perceive and discriminate speech can permeate the academic performance of children with hearing loss. For example, tests of broad reading abilities were administered to patients of medulloblastoma, aged five and older. Specific subsets that were assessed included passage comprehension, reading fluency and letter-word identification. Results demonstrated a steady decline in overall reading ability until five years post-diagnosis in those with severe hearing loss, however not in those without [43]. The same pattern was not observed for maths abilities, however. Although mathematical ability showed the same decline as reading ability, the difference between those with severe hearing loss and those without was not significant. On one hand, this could perhaps highlight that mathematical learning does not rely, to the same extent, on the same verbal or auditory cues as learning language does, but rather symbolic representations and visualisation of numbers. On the other hand, this may reflect choice of statistical analysis. Participants were analysed according to whether they had severe hearing loss (Chang grade ≥ 2b) or did not have severe hearing loss (Chang grade < 2b). There are seven Chang grades and separating them into only two may be an oversimplification. Perhaps distinguishing between them would reveal differences currently hidden. It is also necessary to note that as well as hearing loss, younger age at diagnosis, high risk status and posterior fossa syndrome were also risk factors for declines in academic and intellectual abilities. This illustrates that ototoxicity is not the sole determinant of decline in these measures.

A contrasting cross-sectional study, however, has demonstrated that patients with ototoxicity have impaired reading *and* maths abilities. In a group of 137 childhood survivors of neuroblastoma (mean age of 12 years), those with hearing loss had twice the risk of parent-reported reading and maths difficulties than those without [46]. Furthermore, they had a greater risk of general learning disability and needing special education at school. The children also self-reported dramatically lower scores in the school functioning domain. During the study, survival time was approximately 11 years since diagnosis, which illustrates the long-lasting and damaging repercussions of hearing loss after cancer treatment. This data, however, is based upon subjective parental reports, which challenges the reliability of the findings especially as data on actual school performance were unavailable to corroborate parental opinions. This may explain the discrepant finding regarding maths ability when comparing it to the previous study, which used objective methods [43]. Despite this, decreased school performance has been noted by family members of children with cancer in another study too [45].

These deficits in reading and maths ability can affect academic achievements in adolescence. Cross-sectional research with adults treated for non-CNS tumours, aged nine at diagnosis but aged 31 at follow-up, showed that severe hearing loss was associated with an estimated twofold increased risk of failing to graduate high school (or be unemployed) [33]. Nevertheless, despite these odds, this applied to only 34% of the sample, suggesting that approximately 65% did graduate. This highlights the individual differences and perhaps resilience of children with cancer, and suggests that in some cases, hearing loss may not necessarily lead to decreased opportunities of reaching milestones like this.

Social and emotional difficulties can also arise due to ototoxicity. Compared to children whose hearing remained normal after treatment, those with hearing loss had poorer emotional well-being as reported by parents [47]. Parents were concerned over their child’s mental health, with reports that they felt miserable, frustrated and anxious. They also had reduced independence and a lessened ability to interact and communicate with peers and family—a pattern we have seen before in adults too [26, 29, 31, 33]. These children needed more support during social interactions, which limits the extent to which they can navigate their environment, develop independence and socialise with peers without their parents, both in and out of school. For example, parents were concerned they were not joining in sports and games, were being bullied and were unable to make and maintain friendships. Of course, this must be interpreted with caution as these are the subjective, rather than objective, opinions of parents, not the child. However, in cancer populations specifically there is evidence of good alignment between parent–child ratings of health-related quality of life [48]. Similar findings were also seen in a Swiss sample, whereby children treated for cancer with hearing loss (aged 8–15) had reduced parent-reported physical well-being and impaired peer relationships [49]. This was only true for patients with CNS tumours, however, not non-CNS tumours, where hearing status had no effect on quality of life. This contradicts earlier research, which found hearing loss to negatively affect the quality of life of children with non-CNS cancers [46]. However, this may be attributed to the fact that chronic health conditions frequently found in those with neuroblastoma, which can affect quality of life, were not controlled for in this study [46].

The academic, social and emotional challenges faced by children with hearing loss may lead to a failure to achieve their full potential, for example by having to re-sit school grades, having poorer self-esteem and less social support [50]. As mentioned previously, the role of consistent audiological monitoring cannot be understated. For children this applies to academic, speech and language assessments too, especially as younger age at diagnosis is a risk factor for more severe ototoxicity [51]. Early identification and intervention, for example by providing hearing aids or cochlear implants, could reduce developmental delays. The schools of hearing-impaired children should also be alerted to accommodate the child and ensure their learning is effective, for example by seating the child close to the teacher or perhaps providing one-to-one support. To ensure normative development in children with cancer, attempts should be made to help them remain at a similar social, emotional and academic level as their peers.

## Impacts on employment and everyday life

Hearing loss after chemotherapy has been associated with an increased likelihood of unemployment [33, 44]. It can even effect which job those LWBC believe they are most suited to. Adults treated for cancer who developed severe hearing loss perceived cancer to have a more negative influence on their vocational plans than those without [33]. This shows that for someone who used to live in a hearing world, treatment-induced hearing loss can be career changing. One group whose employment may be particularly impacted by ototoxicity are musicians. Their art relies on hearing pitch and rhythm etc. For this to be impeded creates a fear they will not be able to play again or may be forced to retire prematurely [32]. One musician reported that cancer was less life-changing than the tinnitus they experienced after treatment and mentioned that their hearing loss renders them an unproductive member of society. They even hinted at being better off dead (although this is only one participant, which may not reflect the opinions of others in the study). This emphasizes the extent to which a person’s identity, livelihood and purpose is tied to their career, and for some adults LWBC, ototoxicity has the power to end or change this. It must be noted, however, that this is a study of online healthcare forums, where only those with internet access or, perhaps, who are most concerned about their ototoxicity, contribute to discussion. This may create a biased and potentially unrepresentative sample.

The everyday simple intricacies of life, which no doubt are often taken for granted by healthy individuals, are heavily impacted by the long-term effects of treatment. In general, adults LWBC with ototoxicity have reported a lower quality of life [3, 44]. This is unsurprising given the number of daily activities which can be impeded by hearing loss, tinnitus and poor balance. Although research on the latter is relatively sparse, there is evidence from a cross-sectional study that adults LWBC, with a mean age of 50, have greater imbalance and fear of falling compared to age-matched healthy controls [52]. These two outcomes were comparable to senior controls with a mean age of 70. These results imply that adults who have undergone treatment for cancer are as worried about falling and have balance impairments similar to individuals 20 years older than themselves. These challenges could impair mobility, independence and the general ability to complete daily tasks. This singular study is supported by a scoping and narrative review which investigated the effect of chemotherapy on the balance and movement of people treated for cancer [24, 25]. Similarly, a common finding was that treatment increases postural sway to a greater extent compared to healthy controls, and that this worsens post-treatment [24]. It is not only static balance that becomes impaired, however, but dynamic too. These impairments in balance, which is crucial for countless activities, can make daily life difficult. Seemingly simple tasks, such as walking or climbing the stairs, can become a challenge.

Gait, defined as an individual’s pattern of walking, can also be compromised after chemotherapy. Adults LWBC have demonstrated a slower gait speed and reduced stride length as chemotherapy continues [25]. Impaired gait, as well as balance, have negative implications for safety as it increases the risk of falls, which occur in approximately 30% of survivors [53]. In fact, amongst those receiving neurotoxic therapy (including PBCT), imbalance contributed to over 50% of falls [54]. Furthermore, along with imbalance and impaired gait, risk of falls is associated with poorer quality of life up to five years after treatment [25]. Again, this is unsurprising, since as well as impeding daily life, imbalance and falls can result in fatalities including broken bones, dislocation and head injury. There are methods to improve imbalance, however, such as balance management. This may include rehabilitation of the vestibular system, balance training and given that maintaining balance relies on multiple sensory inputs, protection of other sources of balance information e.g., visual and somatosensory inputs [25, 40]. Monitoring the balance of cancer patients is crucial for early implementation of interventions like these, which could improve quality of life and reduce injury.

Hearing loss and tinnitus also interfere with the simple tasks encountered in everyday life. Compared to adults LWBC who have not developed ototoxicity, those with moderate to severe tinnitus and hearing loss report greater interference in general activity, walking and working. In fact, they also report greater interference in the enjoyment of life [31]. As mentioned previously, the extent of hearing loss and tinnitus does not need to be severe to have a negative impact. Cross-sectional research has demonstrated that adults treated for oropharyngeal cancer who have acquired mild hearing loss and tinnitus are six times more likely to report moderate to severe functional and psychosocial interference than those without hearing loss and tinnitus. This increases to 30 times if the hearing loss and tinnitus are severe [31]. Sleep disturbances, fatigue and lower morning and evening energy have also been associated with ototoxicity in both cross-sectional and qualitative research [29, 44, 55]. Furthermore, amongst previously mentioned challenges related to hearing television and radio, safety issues have been associated with hearing loss, such as being unable to hear doors opening or food cooking [32]. Hearing management interventions, such as hearing aids may improve this, however, they also have drawbacks. For example, one individual described their reduced self-esteem caused by being fitted with a hearing aid at such a young age, which is visible under their short hair [32]. The same person noted that they do not sleep with their hearing aid in, meaning they cannot hear their baby crying at night, which causes distress. Together, this research highlights the everyday difficulties associated with hearing loss, tinnitus and imbalance, which may be unacknowledged by friends, family and clinicians. It is essential for research to investigate which aspects of life are affected by ototoxicity. This is so that specific interventions can be implemented to facilitate people LWBC.

## Mental health impacts

A diagnosis of cancer can place a mental burden on patients [56]. Adding hearing loss, tinnitus and imbalance issues, which patients are often unaware of as a side effect [26, 29, 32] may exacerbate this. However, not everyone is affected the same way. The literature revealed two distinct mindsets when coping with this long-term effect, which, for simplicity, can be categorised into positive and negative emotions. On one hand, some adults are extremely and adversely affected. Cross-sectional research has demonstrated greater interference in mood in adults LWBC with hearing loss and tinnitus compared to those without [31]. Higher levels of stress have also been reported in those with ototoxicity, as well as distress related to imbalance [44] and tinnitus [32]. There is evidence that levels of depression and anxiety are higher in those with hearing loss and tinnitus than those without, which may be reflected in their lower self-reported ratings of quality of life. There were significant differences in the psychological well-being and mental health quality of life subscales, amongst others [44, 55]. Parent-reported mental health issues, such as anxiety and poor emotional well-being, have also been seen in pediatric populations [47]. Together, this research suggests that ototoxicity can affect the mental health of cancer patients from a young age well into adulthood. However, contrasting results from other pediatric populations suggest that children may not always experience mental health issues [46], perhaps highlighting individual differences and the resilience of children with hearing loss.

These impacts on mental health are already burdensome, however they can become more extreme. Qualitative research has shown that adults LWBC with ototoxicity can adopt a suicidal mindset, with one even alluding to overdosing themselves due to the addition of hearing loss to their original diagnosis and chemotherapy [29]. Another spoke more candidly about this, quoting, ‘I’d rather be dead than deaf’, as well as stating that the management of hearing loss and tinnitus are more life-changing and worrying than the cancer itself [32]. Tinnitus has also been reported as controlling and unbearable, as well as a reminder of cancer [26]. At risk of ototoxicity progressing, and quality of life worsening, chemotherapy type or dosage can be altered, however this brings with it the risk of changing the effectiveness of treatment. This presents a difficult trade-off, which is an unfortunate reality faced by many cancer patients. It is of the utmost importance that the mental health status and audio-vestibular functioning of patients are regularly monitored, since interventions (i.e., counselling or hearing aids) could in some cases be lifesaving.

However, not all adults LWBC possess this suicidal mindset. Some, in fact, adopted a survival mindset and expressed gratitude to be alive [26], preferring to be deaf than dead. These individuals perceived hearing loss and tinnitus to be an acceptable price to pay to be cancer free. One even classed them as a ‘souvenir’ and others thought not to worry about them until they were in remission [32]. Generally, these adults adopted a ‘get on with it’ approach, with some learning to live with or even ignore their hearing loss/tinnitus. Interestingly, comparison with others who, in their opinion, experienced more severe side effects made individuals feel lucky that their own side effects were not as bad [26]. This demonstrates an appreciation that their situation could have been worse and contributes to the notion that ototoxicity is an acceptable price to pay to be cancer free. Others compared their ototoxicity to their more severe treatment side effects, which moved hearing loss to a low priority status [29]. Furthermore, those with this survival mindset appeared to display a resilience and motivation to adapt that those with a suicidal mindset did not. For example, some reported using lip reading and written methods of communication, as well as tactical positioning during social scenarios, to ameliorate the effects of hearing loss [26]. It could be suggested that these discrepancies in coping mindsets result from the importance placed on good hearing, which differs from person to person. Some would rather live with ototoxicity because they place greater value on being alive. For others, normal hearing may be perceived as such an essential part of life that they would rather die than live without it. It is important for research to clarify why this discrepancy between coping mindsets exists, as well as identify the risk factors for adopting the life-threatening suicidal mindset. This would help provide support to those who need it most, thus preventing further negative effects on mental health and quality of life. Healthier coping strategies could also be promoted. To our knowledge, the literature is yet to explore why this difference exists, exemplifying a gap in the research which warrants further attention.

Based on the findings of this review, it is evident that further gaps and limitations exist in the literature. Firstly, greater standardisation in the reporting of patient characteristics is required. Most of the included quantitative studies failed to note the range of age at diagnosis. Furthermore, four of them did not report the average age at diagnosis. Given the importance of age to health risks we argue that such reporting needs to be mandatory, especially given that younger age at diagnosis is associated with more severe ototoxicity [51]. There was also a discrepancy between the type of follow up, with some studies reporting the time since diagnosis and others reporting the time since last dose of treatment, again calling for greater standardisation in reporting. Furthermore, of the thirteen quantitative studies, six had a follow-up period of approximately five years or less. Despite a 5-year follow-up being typical for oncology, it could be argued that this should be longer in order to explore the full extent of late effects and to further investigate whether ototoxicity improves or worsen with time. This calls for more longitudinal designs. Additionally, the measurement of tinnitus and balance was often neglected, highlighting a greater need for these to be included in future research alongside hearing loss. Finally, a majority of studies in this review used quantitative designs. Although these provide valuable information, qualitative designs produce richer and more in-depth data on the personal experiences of ototoxicity. The open nature of qualitative studies may help us understand to a greater extent which aspects of life in particular are affected by hearing loss, tinnitus and imbalance, highlighting the need for more studies of this design.

Despite inconsistencies in the literature, it is clear that coping with cancer, ototoxicity and other possible long-term effects of treatment is challenging, distressing and at times, disheartening. It is of an urgent matter to ensure support is offered from multiple disciplines to help people LWBC regain a sense of normality. This may not be the ‘normal’ that they are familiar with, but the tools and the opportunity to create a *new* normal, where they can function and achieve to the same extent as peers, should be provided by healthcare professionals.

## Wider issues

Throughout this review the importance of monitoring ototoxicity in those LWBC has been emphasised. Importantly, a baseline hearing assessment should be conducted (i.e., prior to receiving ototoxic treatment). This prevents clinicians overestimating the prevalence of ototoxicity, since it may be pre-existing due to noise exposure or presbycusis (age-related hearing loss) [21]. It will also help estimate the degree of hearing loss as chemotherapy continues, should it occur. Although this may vary between care providers, there is evidence that a baseline assessment is not always provided. Qualitative research has demonstrated that out of 20 patients LWBC, 19 of them had not been offered a baseline hearing assessment, despite being offered other baseline tests unrelated to hearing [29]. Another qualitative research study found that the number of patients offered a baseline hearing test was similar to the number of patients that had not [32].

This issue has also been explored from the perspective of professionals working within hearing services. One qualitative research study used an online questionnaire to assess the management of patients with ototoxicity in the UK [40]. Respondents were mainly audiologists, audio-vestibular physicians and ear, nose and throat physicians treating patients for cancer, amongst other patient groups. Analysis revealed that only 16% measured baseline hearing and balance, 26% did not and the remainder only tested some patients, but not all. Concerning the subsequent monitoring of ototoxicity, 60% tested hearing loss only, and less than 10% tested for balance only or both. Surprisingly, 30% of respondents did not know whether monitoring was carried out. This highlights that the monitoring of balance is less common than that of hearing loss, which in itself could be improved. It is important to test the balance of individuals LWBC, especially since falls are more common in older adults with cancer than those without [57]. Similar findings have been seen in a study of 17 pediatric oncology centres [58]. A questionnaire sent to each of these centres highlighted the low rate of long-term audiological monitoring. In fact, less than a quarter of participating centres tested children with normal hearing post-treatment. It is important that both children and adults treated with PBCT are monitored long-term, even if they are not presenting with ototoxicity. This is due to the progressive nature of hearing loss even after treatment has ceased [30]. Despite this, there is evidence that some patients, both with and without hearing impairment, have experienced almost a decade between audiological assessments [28].

Failure to monitor ototoxicity during and after treatment can result in a delay in help-seeking. Given the social, cognitive and educational impacts that may arise as a result of ototoxicity, as well as the impacts on child development, employment and mental health, early intervention is crucial. This could include the provision of hearing aids, cochlear implants, counselling or modifying the type/dosage of treatment [40]. To ensure the optimal care of those LWBC, there should be clear communication between oncology and audiology, or even a care team comprised of both. Patients should have routine appointments with audiologists. The American Speech-Language-Hearing Association recommends a hearing assessment 24 hours before each cycle of PBCT and a follow-up at three and six months after the cessation of treatment. For children it is recommended they are assessed immediately after treatment has ended and then at three, six and 12 months [59]. High frequencies should be routinely included in these monitoring assessments as these are the first to deteriorate after PBCT. Despite these guidelines, there is evidence that routine audiological monitoring is not consistently practiced (although this will vary between care providers) [60, 61]. Future research should identify the barriers to conducting baseline measurements and implementing subsequent monitoring programs, with the aim of improving care for those undergoing treatment.

A further problem experienced by those LWBC is the lack of awareness of ototoxicity as a side effect. Qualitative research has found that patients did not know that tinnitus and hearing loss were side effects of cancer treatment until they experienced it themselves [26, 29, 32]. In these three studies, thematic analysis identified the lack of information as a key theme. Many individuals felt as though healthcare professionals had neglected to inform them about ototoxicity, resulting in feelings of disappointment, confusion, anger and dissatisfaction [29, 32]. Others thought that their tinnitus/hearing loss would eventually subside and were shocked when they realised it was permanent. Something to consider, however, is that one of these studies is an analysis of online healthcare forums [32]; therefore, it may be subject to sample bias. Perhaps, forum users most likely to contribute were seen by the healthcare professionals who failed to mention ototoxicity as a side effect, therefore are more likely to express their feelings online. However, even when information on ototoxicity has been provided, there is evidence that it is presented in an inappropriate format (i.e., books and leaflets) at a time when individuals are in shock and have other information to process [29]. This can cause ototoxicity to be overlooked or underestimated as a side effect. More emphasis on this is needed, perhaps through a verbal discussion with a clinician.

The above research highlights the essential role of the clinician, whether that be an audiologist, oncologist or nurse. To enhance the understanding of ototoxicity as a side effect, information should be delivered to the patient in a patient-centred manner, i.e., at a time and in a format tailored to the individual. This could ameliorate the stress, anxiety and shock associated with ototoxicity and the speculation over its permanence. Families of the patients should also be informed to aid their own understanding. Greater information provision may speed up the help-seeking process and prevent negative feelings towards clinicians. It is important that this relationship remains intact, since there is evidence that patients whose oncologist was supportive were more forthcoming about their hearing loss/tinnitus than those whose oncologist was not [29].

## Limitations

It must be noted that although the studies in this literature review all included PBCT, many also included other forms of treatment (e.g., radiotherapy). Therefore, we cannot rule out the possibility that hearing loss, tinnitus and imbalance (and any subsequent negative consequences) were caused by these treatments instead. Furthermore, the studies in this literature review, and in the literature in general, are heterogenous in terms of methodology, participant demographics and measurements of ototoxicity. Therefore, caution must be exercised when making comparisons with regards to incidence and severity. Finally, in this review, screening of the literature was completed by the primary author only. This may reduce how reliable source selection was as there was no second screener to verify the included/excluded papers.

## Conclusion

Although the number of individuals surviving cancer is increasing, the long-term effects of treatment continue to burden several aspects of life for those LWBC. This comprises exclusion and anxiety associated with social scenarios, cognitive difficulties in linguistic and non-linguistic tasks, attention and processing speed. Ototoxicity can affect child development, especially in terms of language acquisition and academic performance. Issues related to impaired audio-vestibular functioning extend into adulthood, with individuals experiencing challenges surrounding the practicalities of everyday life and a greater likelihood of unemployment. Ototoxicity in those LWBC can also have a negative effect on mental health. The risks of depression and anxiety may increase as well as the development of suicidal thoughts. However, not everyone LWBC experiences this. Some have a positive coping mindset and are grateful to have survived even if they have acquired hearing loss or tinnitus. Future research should investigate why this discrepancy exists in order to identify and provide support to individuals with suicidal thoughts, as well as to promote more productive coping strategies.

To reduce these impacts of hearing loss, tinnitus and imbalance early intervention is crucial. However, there is evidence that baseline measurements of ototoxicity and subsequent monitoring is inadequate. Furthermore, many patients felt as though healthcare professionals had not informed them of ototoxicity as a side effect, which caused feelings of anger and distrust. This could cause a breakdown in the patient-clinician relationship. Future research could investigate why these issues exist, which would be beneficial since resolving them could increase the speed at which interventions are sought and may prevent further decline in quality of life. In addition, there is relatively less research on imbalance relative to tinnitus and hearing loss, despite it interfering with daily life and increasing the risk of falls and injury. More research should be dedicated to this long-term debilitating effect of chemotherapy.

Although the aim of cancer treatment is survival, ototoxicity encountered after PBCT, which is often permanent and not preventable, should not be neglected by clinicians and researchers. It is crucial that we direct more of our attention towards this topic to enhance the care of patients and help them adapt to life with ototoxicity. We ought to investigate which aspects of daily life are compromised so that tailored interventions can be applied to situations where help is needed the most. It is the joint responsibility of healthcare professionals and researchers to facilitate this.

### Supplementary Information

Below is the link to the electronic supplementary material.

Supplementary file1 (PDF 68 KB)

## Data Availability

All data analysed are included in this published article (see reference list).
