# Supplementary material for: The long-term impacts of hearing loss, tinnitus and poor balance on the quality of life of people living with and beyond cancer after platinum-based chemotherapy: a literature review
Source: J Cancer Surviv. 2023 Jan 13;17(1):40–58. doi: 10.1007/s11764-022-01314-9 (PMC9971148; doi:10.1007/s11764-022-01314-9)
Supplement: Supplementary file 1 — Supplementary file1 (PDF 68 KB) [file 11764_2022_1314_MOESM1_ESM.pdf]

Supplementary Information

Title: The long-term impacts of hearing loss, tinnitus and poor balance on the quality of life of people living with and beyond cancer after platinum-based chemotherapy: a literature review

Journal: The Journal of Cancer Survivorship

Author names: Olivia R. Phillips[1, 2], David M. Baguley[1, 2], Stephanie E. Pearson [3] & Michael A. Akeroyd[1, 2]

Affiliations

1. Hearing Sciences, Mental Health and Clinical Neurosciences, School of Medicine, University of Nottingham, Nottingham, NG7 2UH, UK
2. NIHR Nottingham Biomedical Research Centre, Ropewalk House, 113 The Ropewalk, Nottingham NG1 5DU, UK
3. The University of Nottingham Health Service, Cripps Health Centre, University Park, Nottingham, NG7 2QW

Email address of corresponding author: michael.akeroyd@nottingham.ac.uk

Online Resource 1: Search strings

Web of Science

(TS=((cancer AND survivor OR "cancer survivor" OR "long term cancer survivor*") AND (chemotherapy AND platinum OR cisplatin OR carboplatin OR oxaliplatin) AND (ototoxic* OR vestibulotoxicity OR "hearing loss" OR tinnitus OR balance OR "long term" OR "quality of life")) AND AB=((cancer AND survivor OR "cancer survivor" OR "long term cancer survivor*") AND (chemotherapy AND platinum OR cisplatin OR carboplatin OR oxaliplatin) AND (ototoxic* OR vestibulotoxicity OR "hearing loss" OR tinnitus OR balance OR "long term" OR "quality of life")))

We used the filter feature on Web of Science to exclude irrelevant research areas e.g., physics. The following research areas were included: Oncology or Paediatrics or Neurosciences Neurology or General Internal Medicine or Otorhinolaryngology or Health Care Sciences Services or Rehabilitation or Audiology Speech Language Pathology

PubMed

((cancer AND survivor OR "cancer survivor") AND (chemotherapy AND platinum OR cisplatin OR carboplatin OR oxaliplatin) AND (ototoxic* OR vestibulotoxicity OR "hearing loss" OR tinnitus OR balance) AND ("long term" OR "quality of life"))

Google Scholar

(cancer AND (survivor OR "cancer survivor" OR "long term cancer survivor")) AND (chemotherapy AND platinum OR cisplatin OR carboplatin OR oxaliplatin) AND (ototoxic* OR vestibulotoxicity OR "hearing loss" OR tinnitus OR balance OR "long term" OR "quality of life")

We found thousands of titles from Google Scholar to be irrelevant after inputting the above search string. Therefore, we decided to extract all the titles using the software ‘Publish or Perish’ to filter these results using a second search. To ensure titles included the relevant population, we filtered them so that only those with the words, “cancer” AND “survivor” OR “cancer survivor” OR “long term” OR “long term cancer survivor” in the titles were retained.
